# Metabolic Response to XD14 Treatment in Human Breast Cancer Cell Line MCF-7

**DOI:** 10.3390/ijms17101772

**Published:** 2016-10-24

**Authors:** Daqiang Pan, Michel Kather, Lucas Willmann, Manuel Schlimpert, Christoph Bauer, Simon Lagies, Karin Schmidtkunz, Steffen U. Eisenhardt, Manfred Jung, Stefan Günther, Bernd Kammerer

**Affiliations:** 1 Center for Biological Systems Analysis ZBSA, Albert-Ludwigs-University Freiburg, 79104 Freiburg, Germany; daqiang.pan@mail.zbsa.uni-freiburg.de (D.P.); Michel.Kather@merkur.uni-freiburg.de (M.K.); lucas.willmann@mail.zbsa.uni-freiburg.de (L.W.); manuel.schlimpert@sgbm.uni-freiburg.de (M.S.); christoph.bauer@pluto.uni-freiburg.de (C.B.); simon.lagies@uranus.uni-freiburg.de (S.L.); 2 Institute of Pharmaceutical Sciences, Albert-Ludwigs-University Freiburg, 79104 Freiburg, Germany; karin.schmidtkunz@pharmazie.uni-freiburg.de (K.S.); manfred.jung@pharmazie.uni-freiburg.de (M.J.); stefan.guenther@pharmazie.uni-freiburg.de (S.G.); 3 Institute of Biology II, Albert-Ludwigs-University Freiburg, 79104 Freiburg, Germany; 4 Spemann Graduate School of Biology and Medicine, Albert-Ludwigs-University Freiburg, 79104 Freiburg, Germany; 5 Department of Plastic and Hand Surgery, University of Freiburg Medical Center, 79106 Freiburg, Germany; steffen.eisenhardt@uniklinik-freiburg.de; 6 BIOSS Centre for Biological Signalling Studies, Albert-Ludwigs-University Freiburg, 79104 Freiburg, Germany

**Keywords:** XD14, 4-acyl pyrrole derivative, Michigan Cancer Foundation-7 (MCF-7), GC-MS, metabolic profiling, cancer therapy, BRD-related tumors

## Abstract

XD14 is a 4-acyl pyrrole derivative, which was discovered by a high-throughput virtual screening experiment. XD14 inhibits bromodomain and extra-terminal domain (BET) proteins (BRD2, BRD3, BRD4 and BRDT) and consequently suppresses cell proliferation. In this study, metabolic profiling reveals the molecular effects in the human breast cancer cell line MCF-7 (Michigan Cancer Foundation-7) treated by XD14. A three-day time series experiment with two concentrations of XD14 was performed. Gas chromatography-mass spectrometry (GC-MS) was applied for untargeted profiling of treated and non-treated MCF-7 cells. The gained data sets were evaluated by several statistical methods: analysis of variance (ANOVA), clustering analysis, principle component analysis (PCA), and partial least squares discriminant analysis (PLS-DA). Cell proliferation was strongly inhibited by treatment with 50 µM XD14. Samples could be discriminated by time and XD14 concentration using PLS-DA. From the 117 identified metabolites, 67 were significantly altered after XD14 treatment. These metabolites include amino acids, fatty acids, Krebs cycle and glycolysis intermediates, as well as compounds of purine and pyrimidine metabolism. This massive intervention in energy metabolism and the lack of available nucleotides could explain the decreased proliferation rate of the cancer cells.

## 1. Introduction

Breast cancer is the most frequent cause of cancer death among women. The incidence of female breast cancer has been continually rising in recent years [1,2,3,4]. However, the mortality rate is decreasing in most developed countries due to the development of early diagnosis methods and therapies [1,2].

**Figure 1:**
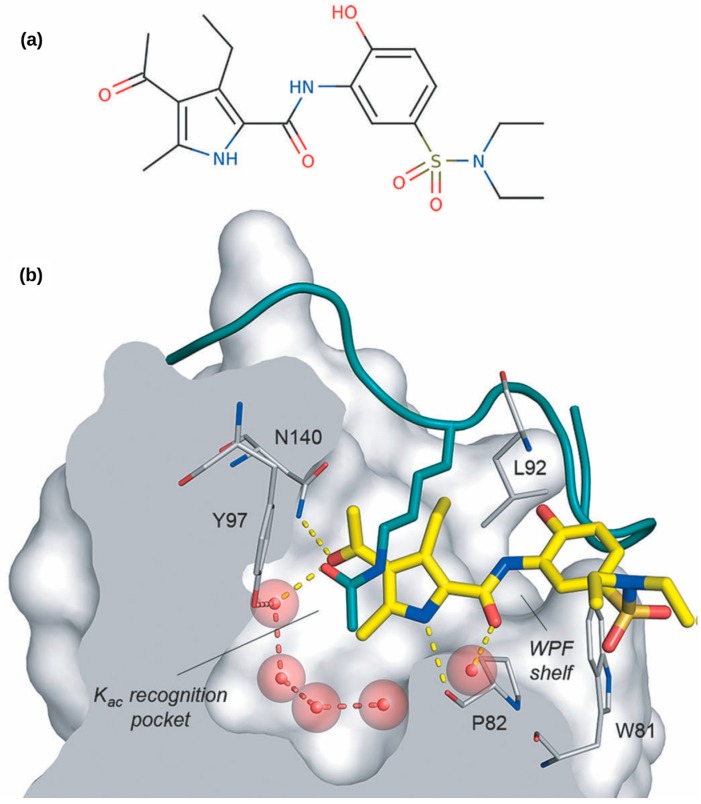
(**a**) Chemical structure of XD14; (**b**) XD14 (yellow sticks) inhibits the recognition of the acetyl-lysine of histone (green sticks) by BRD4. Important interacting residues of BRD4 are shown in grey. Hydrogen-bonds are shown as discontinuous lines [5]. Copyright Wiley-VCH Verlag GmbH & Co. KGaA. Reproduced with permission.

In this work, a new drug, XD14 (chemical name: 4-acetyl-*N*-[5-(diethylsulfamoyl)-2-hydroxyphenyl]-3-ethyl-5-methyl-1*H*-pyrrole-2-carboxamide (Figure 1a)), was applied to human breast cancer cell line MCF-7. The inhibitor XD14 was discovered in a high-throughput virtual screening experiment [5]. It binds the first bromodomain (BRD) (Figure 1b) of BRD4 from the bromodomains and extra-terminal (BET) subfamily with a dissociation constant (*K*_D_) of 237 nM.

Bromodomains, containing about 110 amino acids, are protein domains that are found in diverse nuclear protein complexes, including ATP-dependent chromatin-remodeling complexes, helicases, methyltransferases, transcriptional coactivators, transcription factors, and nuclear-scaffolding proteins [6]. As “histone readers”, BRDs can specifically recognize acetyl-lysines (*K*_ac_) in the N-terminal regions of H3 and H4 of histones [7,8]. BRDs, in concert with other protein-interaction modules, ensure highly specific recognition and binding of chromatin-modifying enzymes. There are 61 BRDs encoded in the human genome clustering into eight families based on structure and sequence similarity [6]. All BRDs share a conserved fold containing four α-helices linked by three different loops, which define the *K*_ac_ binding site and determine binding specificity. Since BRDs take part in transcription regulation, they have been applied as potential therapeutic targets in research of diverse cancer types, inflammation, and some other diseases [9,10,11,12].

BET inhibitors have been developed for tumor related research in recent years [13]. JQ1 [14] and I-BET151 [15] have shown potential therapeutic effects against acute myeloid leukemia and mixed lineage leukemia, respectively. Additionally, they are both in preclinical development. XD14 has been applied to 59 different human tumor cell lines, showing variable inhibition of proliferation [5]. As a result of histone reader inhibition, some cancer related genes were down-regulated [15,16]. However, investigation of the metabolic signature has not been covered in these studies.

Metabolic profiling has been applied in different fields of research including characterization of plants, bacteria, and cancer as it reflects the actual phenotype of a system [17,18,19,20,21,22,23]. The alteration of metabolites could be a result of genetic, pathological, or environmental changes. As reported, many metabolite levels were altered in the blood plasma and urine of cancer patients or cancer cell lines [24,25,26,27,28,29,30,31]. Metabolic profiling can reveal the alteration of metabolite levels in cancer patients, and, consequently, novel biomarkers could be discovered [32]. Therefore, metabolic profiling might be beneficial for the understanding of molecular processes induced by XD14 treatment in cancer cells.

Through the application of metabolic profiling in this study, massive alterations of metabolic pathways including central energy metabolism, amino acid metabolism, and fatty acid metabolism were observed; this gives a novel aspect to the application and evaluation of XD14 treatment.

## 2. Results

### 2.1. Inhibition of Cell Proliferation after Treatment with XD14

**Figure 2:**
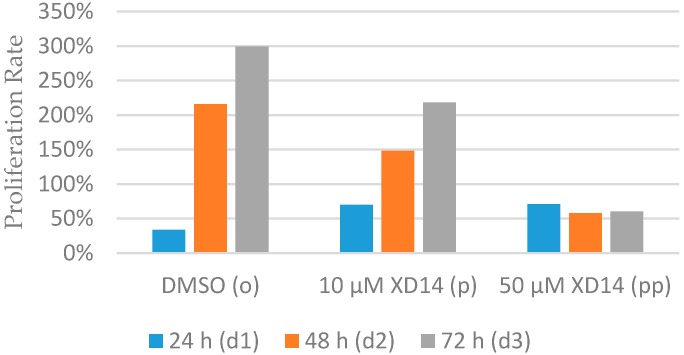
Proliferation rate of MCF-7 cells after treatment with DMSO (group o) or XD14 (group p and pp) for 24, 48, and 72 h. The proliferation rate was calculated based on the cell number of d (day)1, d2 and d3 compared to d0.

Cell proliferation was evaluated by cell number in each condition (all cell numbers are shown in Table A1). As shown in Figure 2, cell proliferation rates (proliferation rate = 100 × (cell number_present_ − cell number_initial_)/cell number_initial_) in group o (treated with DMSO (Dimethyl sulfoxide)) were around 200% and 300% for 48 and 72 h, respectively, while in group p (treated with 10 μM XD14) they were around 150% and 200%, respectively. Surprisingly, the cell proliferation rate remained around 50% in group pp (treated with 50 μM XD14), indicating an obvious inhibition of cell proliferation by 50 µM XD14 treatment.

### 2.2. Global Metabolic Profile and Statistical Evaluation

Around 500 mass spectral features could be found in each sample including metabolites, artifacts, and background. Seven hundred forty-seven features remained in the output peak intensity matrix of overall corresponding quantifier ions after processing with AMDIS (Automated Mass Spectrometry Deconvolution and Identification System) [33] and SpectConnect [34]. By application of mass spectral databases (NIST, Golm, Fiehn) 117 metabolites were identified.

Seventy-six metabolites were significantly (*p* < 0.05) altered based on analysis of variance (ANOVA). False discovery rate (FDR) was applied to find the false significant metabolites in multiple comparisons. After correction, the number of significant metabolites was reduced from 76 to 67 by application of a threshold of 0.05 as well. The significantly altered metabolites belong to amino acids; lipids/phospholipids and their derivatives; glycolysis and tricarboxylic acid (TCA) intermediates; purine and pyrimidine metabolism; and others (Table A2).

**Figure 3:**
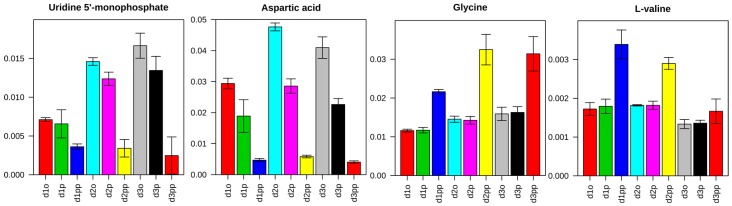
Example of regulated metabolites in control and two treatment groups at three time points. Uridine 5´-monophosphate (UMP) and aspartic acid showed correlated down regulation with treatment concentrations while glycine and valine showed significant up regulation only in the 50 µM XD treatment groups.

Individual metabolites showed different regulation patterns among groups. For instance, four interesting metabolites are shown in Figure 3. Uridine 5´-monophosphate (UMP) and aspartic acid showed more reductions with 50 µM treatment than with 10 µM, which correlates to the concentrations of treatment. However, glycine and valine showed significant up regulation with only the 50 µM treatment. Since no consistent regulation patterns were found in individual metabolites, further discrimination of the groups is essential with other statistical methods.

**Figure A1:**
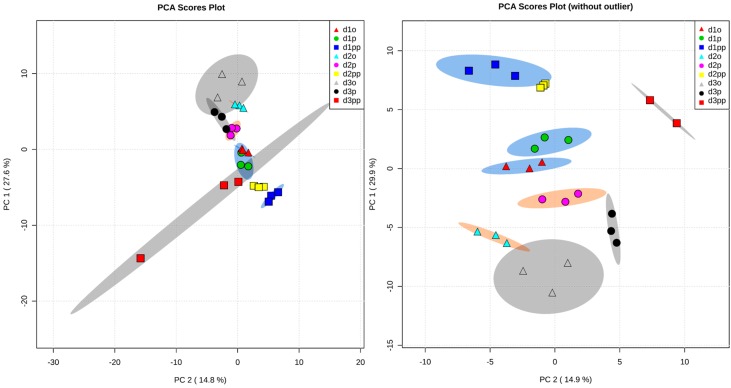
PCA scores plots before (**left**) and after (**right**) removal of outlier. After removal of outlier, all groups could be separated with PCA.

After evaluation with principal component analysis (PCA), an outlier was detected in group d3pp (Figure A1), which was removed from the data set. The reason for the outlier was certainly an instrumental error. As seen in the sample list (Table A3), the first analyzed sample was d3pp_1, which was the outlier. It could be that the column was not completely equilibrated and, consequently, the first analysis was not reliable. After removal of the outlier, all sample groups could be separated by PCA as shown in Figure A1.

**Figure 4:**
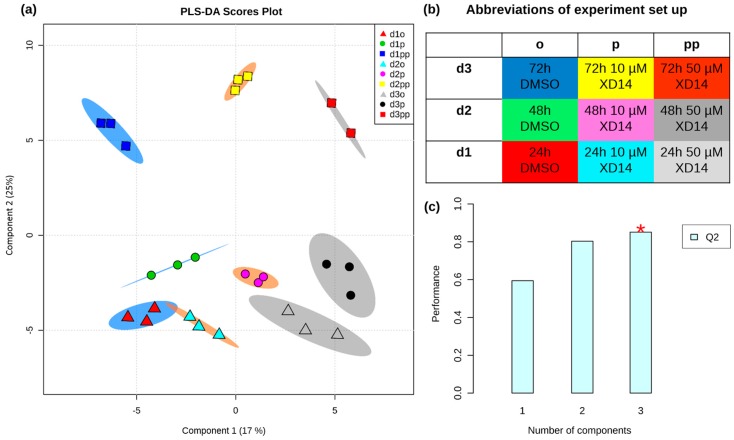
(**a**) Partial least squares discriminant analysis (PLS-DA) scores plot discriminates all samples based on time (day 1, 2, and 3 are in blue, orange, and grey shaded brackets, respectively) in component 1 and concentration (control, 10, and 50 µM XD14 treated groups are in triangle, circle, and square, respectively) in component 2. The explained variances of each group are shown in brackets; (**b**) Abbreviation of experiment setup; (**c**) Classification performance using different numbers of components, showing the explained variance in prediction Q^2^. The red asterisk indicates the best classifier.

A scores plot was generated in Figure 4a by partial least squares discriminant analysis (PLS-DA), showing clear discrimination of all sample groups by time (component 1) and concentration (component 2) of treatment. The abbreviations of experiment set up are shown in Figure 4b. All triplicates are clustered together, indicating a high degree of similarity for each triplicate. Both concentrations of treatments showed different effects that discriminated them from the control sample. The samples treated with 50 µM XD14 have a positive component 2 while the samples with low concentration and controls both have a negative component 2. This indicates that treatment with 50 µM XD14 had a stronger effect on metabolite levels than treatment with 10 µM XD14. The prediction performance was evaluated by cross validation using different numbers of components. Q^2^ indicates predictability with an optimal value of 1 [35]. Although three components achieved the best performance, Q^2^ with two components provided a sufficient prediction (Figure 4c).

### 2.3. Alterations of Metabolic Profile after Two Days of 50 µM XD14 Treatment

**Figure 5:**
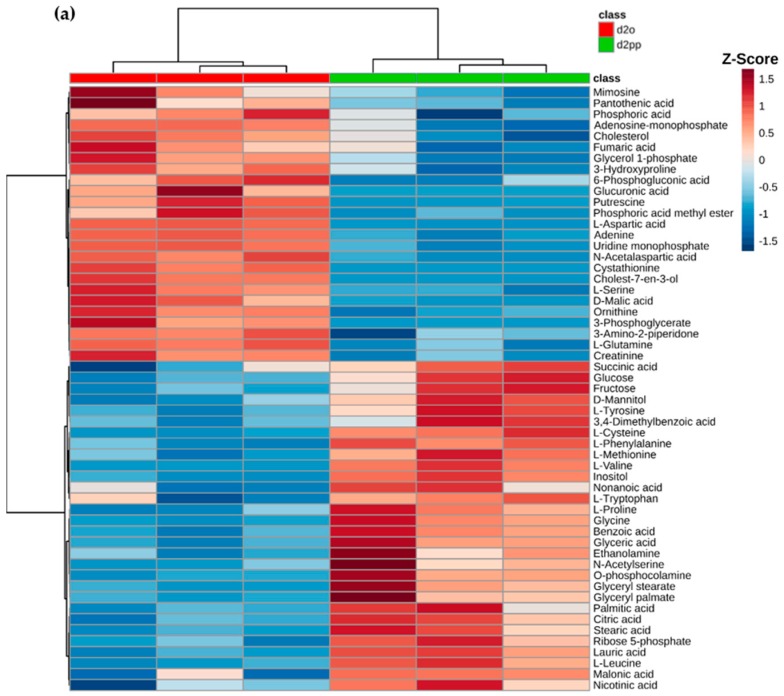

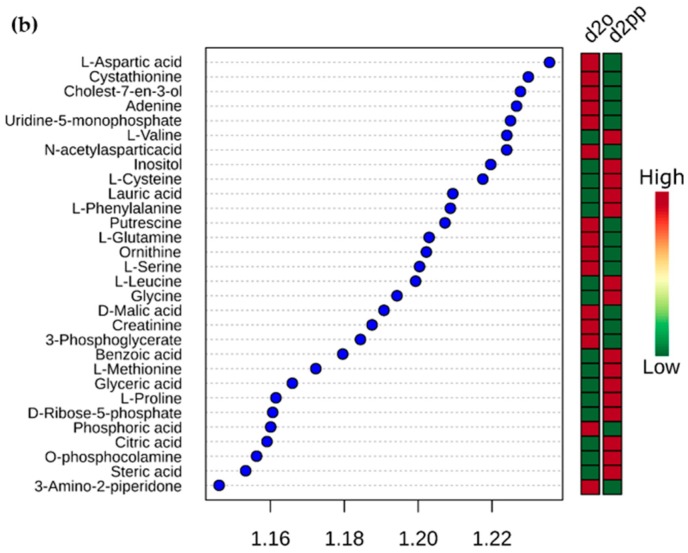
(**a**) Heat map of the metabolic profile in groups d2o and d2pp (*n* = 3) after XD14 treatment for two days; (**b**) Top significant metabolites based on variable importance in projection (VIP) score of component 1 in PLS-DA. Colored boxes indicate the relative concentrations of corresponding metabolites.

The cell proliferation rate of control is four times reduced after 50 µM XD14 treatment for two days (Figure 2), indicating an obvious inhibition of cell proliferation. Therefore, the metabolic profile at day 2 was analyzed and considered as the response to the XD14 treatment. A heat map of metabolites was generated based on the data of group d2o and d2pp, showing an altered metabolic profile in the treated group compared to control group (Figure 5a). Z-score was calculated by dividing mean-centered peak areas with standard deviation of each variable.

The variable importance in projection (VIP) [35] in PLS-DA shows the important metabolites, which explains most variation between the two groups. Eleven out of 30 important metabolites are amino acids or their derivatives (Figure 5b), which were mainly investigated in PLS-DA to discriminate treated from non-treated breast cancer cells.

#### 2.3.1. Pathway Analysis of Significantly Regulated Metabolites

**Figure 6:**
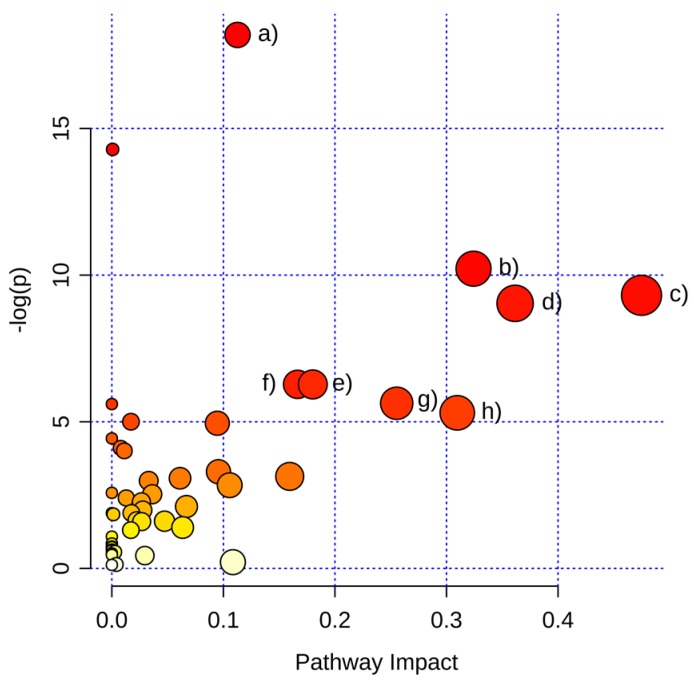
Map of pathway analysis shown in pathway impact and −log(p). Pathway impact is the pathway impact value calculated from pathway topology analysis [36]. (a) Aminoacyl-tRNA biosynthesis; (b) Glycine, serine, and threonine metabolism; (c) Alanine, aspartate, and glutamate metabolism; (d) Arginine and proline metabolism; (e) Pantothenate and CoA biosynthesis; (f) Phenylalanine metabolism; (g) Pentose phosphate pathway; (h) Cysteine and methionine metabolism.

Twelve pathways were significantly changed by a pathway analysis (Table A4). Six of them are amino acid metabolism pathways, indicating a massive alteration of amino acids biosynthesis and metabolism (Figure 6). This could be a result of the change in energy metabolism (TCA cycle), which has an influence on the biosynthesis/metabolism of some amino acids. Moreover, a changed cell proliferation resulted in an altered amino acid demand for protein biosynthesis. The alteration in pantothenate and CoA biosynthesis resulted in a dysregulation of CoA synthesis, which had an effect on fatty acid biosynthesis/metabolism and oxidation of pyruvate in citrate (the starting point of the TCA cycle). The regulation of glutathione metabolism may be a result of oxidative stress caused by XD14 treatment. The dysregulation of the pentose phosphate pathway may be a result of the inhibition of cell proliferation and, consequently, reduced the biosynthesis of ribose.

#### 2.3.2. Change in Amino Acid Metabolism

**Figure 7:**
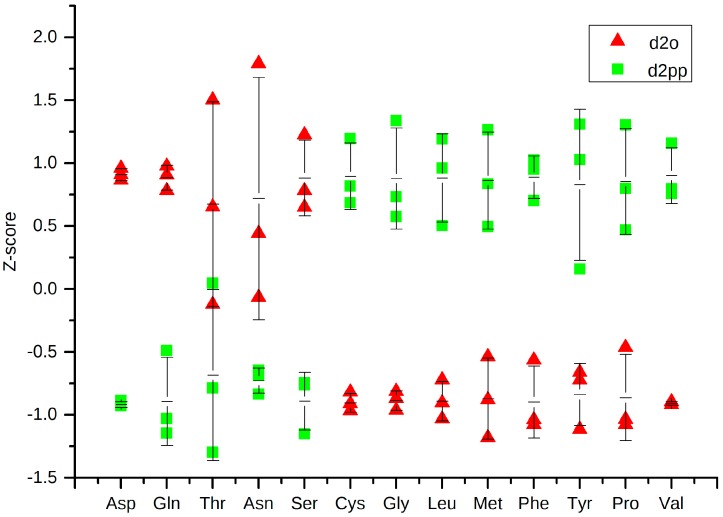
Dot plot of amino acid levels in groups d2o and d2pp, showing the z-scores with average and error bars of each substance.

**Figure 8:**
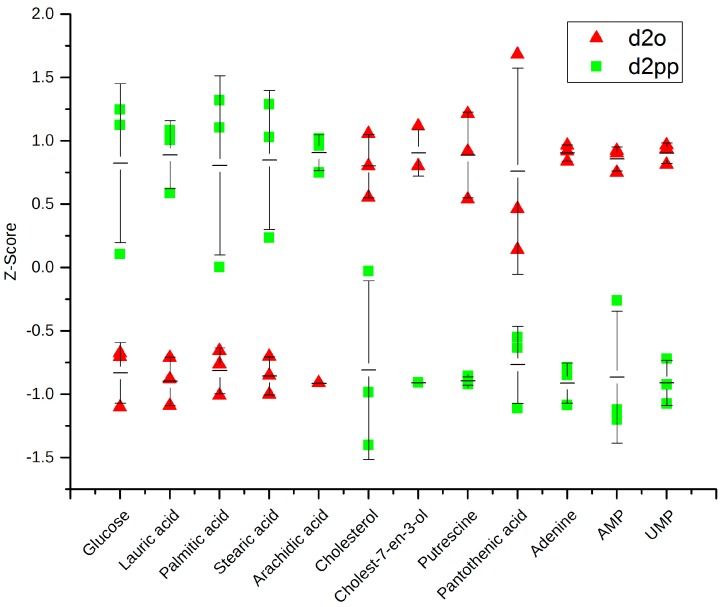
Dot plot with average and error bars of glucose; four free fatty acids; cholesterol and its derivative; putrescine; pantothenate; adenine, UMP and adenosine monophosphate (AMP) in groups d2o and d2pp.

Five out of 17 of the found amino acids were down-regulated after treatment, while nine were up-regulated. A decrease of most amino acid levels has been reported in the blood plasma of breast cancer patients [28] and different breast cancer cell lines [29]. Assuming elevated energy consumption and consequent channeling of amino acids into the TCA cycle, Val, Tyr, Phe, Trp, Met, and Leu were decreased in breast cancer in both of the two studies. After treatment with XD14, these amino acids remained in higher levels compared to non-treated cells (Figure 7). Meanwhile, the glucose level was higher in XD14 treated cells as well (Figure 8), indicating a lower energy consumption after treatment. However, Asp, Gln, Thr, Asn, and Ser were down-regulated after treatment.

#### 2.3.3. Dysregulations of Other Metabolites

As described in pathway analysis, pantothenate and CoA biosynthesis was significantly changed after XD14 treatment. Pantothenate was down-regulated (Figure 8), indicating an inhibited biosynthesis of CoA. Given that cell proliferation was inhibited in treated cells, the consumption of fatty acids for membrane production decreased and resulted in accumulation of free fatty acids (Figure 8). Besides, glucose was up-regulated while putrescine, adenine, UMP, and adenosine monophosphate (AMP) were down-regulated.

## 3. Discussion

To the best of our knowledge, this study is the first time that metabolomic techniques were applied to monitor metabolic effects of a BET inhibitor treated cancer cell line. Metabolic disturbances were revealed by GC-MS, showing changes, for example, in amino acid metabolism and fatty acid metabolism. These changes provide evidence of inhibited cell proliferation and can be taken into account in the cellular response to epigenetic regulation caused by XD14.

Cell proliferation was strongly inhibited after treatment with 50 µM XD14. Although XD14 has a low dissociation constant (*K*_D_ = 237 nM), 10 µM XD14 showed only a slight inhibition of proliferation; this could be caused by a low diffusion constant of XD14 through the cellular membranes. Therefore, a structural optimization of this molecule may be important for a lower concentration of treatment. Based on the structure of XD14, Hügle et al. [37] have done a series of modifications, which increased the target specificity of inhibitors to different BRDs. However, the diffusion rates through cellular membranes of these molecules remain to be tested.

GC-MS analysis yielded robust and reproducible analyses for each metabolite feature. A randomized sample order (Table A3) was applied to prevent possible instrument drift causing false “significant” differences. Despite that, biological replicates yielded comparable peak areas. This can be observed in clustering of triplicates in PLS-DA (Figure 4a) and small standard deviations in dot plots (Figure 7 and Figure 8). Multivariate data analysis revealed significant differences between groups with different concentrations of XD14 treatment and with different sampling time using PLS-DA. They showed that component 1 was influenced by different sampling times and component 2 was influenced by the concentration of the XD14 treatment. The most significant differences were observed with the 50 µM XD14 treatment after two days of incubation. Consequently, further analysis was focused on this concentration condition (Figure 5a).

Twelve metabolic pathways including aminoacyl-tRNA biosynthesis; nitrogen metabolism; glycine, serine, and threonine metabolism; alanine, aspartate, and glutamate metabolism; arginine and proline metabolism; phenylalanine metabolism; pantothenate and CoA biosynthesis; pentose phosphate pathway; cyanoamino acid metabolism; cysteine and methionine metabolism; glutathione metabolism; and citrate cycle were significantly changed (Table A4). Surprisingly, six of the altered pathways were amino acid metabolism pathways. Many amino acids remained at higher levels after treatment indicating lower energy consumption than non-treated tumor cells. This could also explain the higher level of glucose in the treated cells. Jain et al. [24] suggested that glycine plays a key role in rapid cancer proliferation, while Labuschagne et al. [38] proposed serine rather than glycine. In our study, serine was down-regulated while glycine was up-regulated, which correlates with the results of Labuschagne et al. The reduction of serine in treated cells could be a result of epigenetic regulation caused by XD14 which might have down-regulated serine hydroxymethyl transferase (SHMT). Therefore, glycine could not be converted to serine and consequently glycine was accumulated. Besides, Labuschagne et al. showed that serine is an essential precursor for purine nucleotides synthesis. This explains the reduction of adenine and AMP (Figure 8). By the identification of massive alterations of amino acid concentrations in this study, the application of amino acids could be beneficial in future tumor diagnostic.

Inhibited cell proliferation and consequently decreased fatty acid consumption for membrane biosynthesis could lead to an accumulation of free fatty acids. Furthermore, reduced a concentration of pantothenic acid indicates a lower abundance of CoA and thus leads to reduced migration of free fatty acids over the mitochondrial membranes for β-oxidation. Decreased cholesterol and its derivative levels suggested a reduced biosynthesis of the tumor-related hormones estrogen and progesterone. Cholesterol is a precursor for biosynthesis of estrogen and progesterone [39], which are important factors in breast cancer development [40,41]. As a result of XD14 treatment, the levels of cholesterol and its derivative cholest-7-en-3-ol were decreased (Figure 8), indicating a reduced production of estrogen and progesterone. Consequently, the cell proliferation was inhibited.

Putrescine, spermidine, spermine, and cadaverine are polyamines, which are suggested to be essential for cell cycle and proliferation. Cells with a low concentration of polyamines are arrested in the cell cycle [42,43,44,45]. After XD14 treatment, the concentration of putrescine was significantly decreased (Figure 8). This gives evidence to the possible arrest of cell proliferation.

BET inhibitors have been in discussion in regard to tumor related research [13]. In consideration of reduced proliferation, which is reflected by detected metabolite concentrations, our results suggest a great potential of XD-14 in the treatment of breast cancer. Especially, the decreased amino acid metabolism which has been highlighted in our study rather reflects a healthy energy status of the cell. Consequently, the results of this in vitro study should be transferred to in vivo studies for proof of concept because cell line analyses exhibit static, non-physiological conditions, and disregard, for example, the tumor microenvironment.

## 4. Materials and Methods

### 4.1. Cell Culture and XD14 Treatment

MCF-7 cells were cultured in RPMI 1640 medium (PAN Biotech, Aidenbach, Germany) with 2 mM l-glutamine (PAN Biotech, Aidenbach, Germany) and 10% FCS (Sigmal-Aldrich, Munich, Germany) at 37 °C and 5% CO_2_. XD14 was resolved in DMSO (Fluka, Munich, Germany) as stock solution in 10 and 50 mM.

Cells were plated in 10 cm petri dishes at a cell density of 1.33 × 10^6^ for a series experiments for 24, 48, or 72 h. After incubation of 24 h for recovery, the medium was removed and fresh medium with DMSO, 10 μM XD14, or 50 μM XD14 was added. Each condition was applied in four replicates, from which three were applied for metabolic profiling and one for cell number counting. The end concentration of DMSO in every cell culture was 0.1% (*v*/*v*). The cells were counted using Neubauer hemocytometer (Paul Marienfeld GmbHCo.KG, Lauda-Königshofen, Germany) after 3 min trypsin/EDTA (PAN Biotech, Aidenbach, Germany) incubation.

### 4.2. Metabolite Extraction

The cells were washed once with 10 mL 0.9% NaCl in ultrapure water to remove detached cells. 1 mL 20 °C methanol/water (*v*/*v*: 90/10) containing 1 μg/mL of phenyl β-d-glucopyranoside (Sigmal-Aldrich, Munich, Germany) as internal standard was transferred to the cell culture. Following the method of Willmann et al. [29], direct methanolic extraction was applied. Petri dishes were immediately placed on ice and cells were detached with a cell lifter. Cell suspensions were transferred into 2 mL screw-cap tubes, which were prefilled with 300 mg glass beads (diameter 425–600 μm; Sigma, Munich, Germany) and then frozen in liquid nitrogen until homogenization.

Screw-cap tubes were put into a Precellys tissue homogenizer (Bertin Technologies, France) and cell disruption was performed by three 15 s operating cycles at −10 °C and a maximum intensity with a 10 s break between each cycle. The metabolite-containing supernatant was transferred to a new reaction tube (Eppendorf, Hamburg, Germany) after centrifugation at 21,000× *g* and 4 °C for 10 min. In order to reduce the loss of samples, the screw-cap tubes were retreated with 1 mL extraction medium and the supernatant was transferred to the same reaction tube after a short homogenization with a vortex mixer (Eppendorf, Hamburg, Germany) and centrifugation. Supernatant was dried in a concentrator plus vacuum rotator (Eppendorf, Hamburg, Germany) and stored under nitrogen atmosphere at −80 °C until derivatization.

### 4.3. Derivatization and GC-MS Analysis

According to the protocol of Fiehn [46], samples were warmed up at room temperature for at least 15 min before opening. Derivatization was done by shaking with 20 μL 20 mg/mL methoxyamine (Sigma–Aldrich, Munich, Germany) in pyridine (Sigma–Aldrich, Munich, Germany) at 1200 rpm and 28 °C for 90 min and after centrifugation at 14,000 rpm for 30 s with 50 μL *N*-methyl-*N*-trimethylsilyltrifluoroacetamide (Sigma–Aldrich, Munich, Germany) at 1200 rpm and 37 °C. Sample solutions were transferred to glass vials (VWR, Darmstadt, Germany) for GC-MS analysis.

The following operations were performed before GC-MS analysis: an autotune operation for optimal parameters, the mass calibration using PFTBS, and a C10-C40 *n*-alkane standard mixture (Neochema, Bodenheim, Germany) for retention index calculation. The samples were analyzed using randomized block design (Table A3). A 1 μL portion of each sample was injected in splitless mode into an Agilent 7890A/5975C system equipped with a Gerstel MPS 2 XL autosampler. A HP-5MS capillary column with the dimension 29.185 m × 0.25 mm × 0.25 μm was used for GC separation, during which helium was used as a carrier gas. The applied temperature program started at 80 °C for the first two min and was heated up to 320 °C over 50 min and kept at 320 °C for 10 min.

### 4.4. Data Processing and Statistical Evaluation

Spectra of all GC-MS analysis were processed using AMDIS (Version 2.71, National Institute of Standards and Technology, Gaithersburg, MD, USA) and NIST MS Search 2.0 (National Institute of Standards and Technology, Gaithersburg, MD, USA) with the following parameters: 12 component width, adjacent peak subtraction one, resolution medium, sensitivity medium, and shape requirement medium. AMDIS produced for each data file an *.ELU file as output, which was uploaded to SpectConnect to find conserved metabolites in the samples. The data of all the conserved metabolites was then annotated according to the retention index and match score in NIST [47], GolmDB [48], and FiehnLib [49] databases. The maximal retention index variance was 5% and the minimal match score 750. The remaining metabolites were analyzed by MetaboAnalyst 3.0 [50] with normalization by internal standard and peak area sum of each sample. An auto scaling was applied by dividing mean-centered peak areas with a standard deviation of each variable. The following statistical analyses were performed: ANOVA with a *p*-value threshold 0.05 and afterwards FDR with a *p*-value threshold 0.05; PCA, PLS-DA with cross validation using leave one out cross validation; and hierarchical cluster analysis according to Pearson and Ward. Significantly changed metabolites were applied to a pathway analysis by MetaboAnalyst based on the KEGG database.

## Appendix A

Cell number of each group after treatment, identified significantly altered metabolites, order of sample injection and significantly altered pathways are shown in Table A1, Table A2, Table A3 and Table A4.

**Table A1:** Cell number of MCF-7 culture after treatment with DMSO (group o) or XD14 (group p and pp) for 0, 24, 48 and 72 h.

Group\Time	0 h (d0)	24 h (d1)	48 h (d2)	72 h (d3)
o	1.33 × 10^6^	1.78 × 10^6^	4.20 × 10^6^	5.31 × 10^6^
p	1.33 × 10^6^	2.26 × 10^6^	3.30 × 10^6^	4.23 × 10^6^
pp	1.33 × 10^6^	2.27 × 10^6^	2.10 × 10^6^	2.13 × 10^6^

**Table A2:** Significant altered metabolites divided into different substance classes.

Metabolites	*p* Value	FDR	Metabolites	*p* Value	FDR
*Amino acid*			*Lipid/phospholipid and their derivatives*		
l-Aspartic acid	3.65 × 10^−13^	4.23 × 10^−11^	Cholesterol	4.39 × 10^−4^	1.18 × 10^−3^
l-Serine	5.94 × 10^−8^	6.89 × 10^−7^	Cholest-7-en-3-ol	2.62 × 10^−9^	3.79 × 10^−8^
Glycine	5.65 × 10^−11^	2.18 × 10^−9^	Heptanoic acid	5.83 × 10^−5^	2.05 × 10^−4^
l-Leucine	1.46 × 10^−6^	1.13 × 10^−5^	Stearic acid	2.85 × 10^−4^	8.26 × 10^−4^
l-Methionine	8.54 × 10^−6^	4.31 × 10^−5^	Glyceryl stearate	1.96 × 10^−4^	5.97 × 10^−4^
l-Valine	4.01 × 10^−10^	7.75 × 10^−9^	Palmitic acid	7.78 × 10^−4^	1.88 × 10^−3^
l-Tyrosine	4.41 × 10^−6^	2.56 × 10^−5^	Glyceryl palmate	1.53 × 10^−3^	3.42 × 10^−3^
l-Tryptophan	5.45 × 10^−6^	3.01 × 10^−5^	Myristic acid	5.48 × 10^−3^	1.01 × 10^−2^
l-Glutamine	5.46 × 10^−5^	1.98 × 10^−4^	Lauric acid	5.56 × 10^−3^	1.01 × 10^−2^
l-Cysteine	3.29 × 10^−5^	1.24 × 10^−4^	Nonanoic acid	9.14 × 10^−3^	1.45 × 10^−2^
l-Phenylalanine	2.30 × 10^−4^	6.83 × 10^−4^	Palmitoleic acid	8.26 × 10^−3^	1.39 × 10^−2^
l-Threonine	9.03 × 10^−4^	2.14 × 10^−3^	Arachidic acid	3.43 × 10^−6^	2.21 × 10^−5^
l-Proline	2.61 × 10^−3^	5.32 × 10^−3^	*O*-phosphocolamine	2.19 × 10^−11^	1.27 × 10^−9^
l-Asparagine	2.87 × 10^−3^	5.64 × 10^−3^	Phosphoric acid	7.76 × 10^−4^	1.88 × 10^−3^
l-Glutamic acid	9.92 × 10^−5^	3.20 × 10^−4^	Phosphoric acid monomethyl ester	5.62 × 10^−4^	1.48 × 10^−3^
*N*-acetyl-Aspartic acid	3.31 × 10^−5^	1.24 × 10^−4^	Glycerol 1-phosphate	8.38 × 10^−3^	1.39 × 10^−2^
*N*-acetyl-Serine	2.13 × 10^−6^	1.54 × 10^−5^	Pyrophosphate	5.90 × 10^−3^	1.05 × 10^−2^
4-hydroxy-Proline	2.59 × 10^−5^	1.07 × 10^−4^	*Purine and Pyrimidine metabolism*		
Creatinine	8.50 × 10^−5^	2.90 × 10^−4^	Adenosine-5-monophosphate	3.27 × 10^−5^	1.24 × 10^−4^
Citrulline	1.22 × 10^−3^	2.79 × 10^−3^	Adenine	2.17 × 10^−3^	4.67 × 10^−3^
Ornithine	2.53 × 10^−6^	1.73 × 10^−5^	5-deoxy-5-methylthio-Adenosine	2.27 × 10^−3^	4.78 × 10^−3^
3-Amino-2-piperidone	1.56 × 10^−5^	7.26 × 10^−5^	Guanosine-5-monophosphate	7.73 × 10^−3^	1.32 × 10^−2^
*Glycolysis and TCA intermediates*			Uridine 5′-monophosphate	2.03 × 10^−10^	5.00 × 10^−9^
d-Glucose-6-phosphate	1.73 × 10^−5^	7.45 × 10^−5^	*Others*		
d-Mannose-6-phosphate	1.20 × 10^−5^	5.78 × 10^−5^	Malonic acid	1.73 × 10^−5^	7.45 × 10^−5^
d-Gluconic acid	9.51 × 10^−10^	1.58 × 10^−8^	Aminomalonic acid	6.90 × 10^−4^	1.74 × 10^−3^
3-Phosphoglycerate	1.74 × 10^−8^	2.25 × 10^−7^	Taurine	5.06 × 10^−3^	9.63 × 10^−3^
d-Malic acid	8.62 × 10^−8^	8.39 × 10^−7^	Pantothenic acid	5.87 × 10^−7^	5.24 × 10^−6^
Lactic acid	1.35 × 10^−6^	1.12 × 10^−5^	Threonic acid	6.39 × 10^−3^	1.12 × 10^−2^
Citric acid	6.19 × 10^−6^	3.26 × 10^−5^	Propanediol	5.43 × 10−3	1.01 × 10^−2^
Fumaric acid	3.99 × 10^−4^	1.10 × 10^−3^	d-Threitol	8.84 × 10^−5^	2.93 × 10^−4^
Glyceric acid	6.68 × 10^−4^	1.72 × 10^−3^	Inositol	1.01 × 10^−3^	2.34 × 10^−3^
Glycolic acid	2.81 × 10^−3^	5.61 × 10^−3^	Cystathionine	2.15 × 10^−10^	5.00 × 10^−9^
Glucose	3.42 × 10^−3^	6.61 × 10^−3^	Putrescine	8.68 × 10^−8^	8.39 × 10^−7^
Fructose	8.51 × 10^−3^	1.39 × 10^−2^			
6-Phosphogluconic acid	4.31 × 10^−6^	2.56 × 10^−5^			

**Table A3:** Order of sample injection.

Order	Sample	Order	Sample	Order	Sample	Order	Sample
1	d3pp_1	8	d2p_1	15	d3o_2	22	d3p_2
2	d2pp_2	9	d2o_2	16	d1p_2	23	d3pp_3
3	d2pp_1	10	d1pp_2	17	d3p_1	24	d2p_3
4	d2pp_3	11	d2o_3	18	d3o_3	25	d1o_3
5	d1pp_1	12	d3o_1	19	d1p_3	26	d1pp_3
6	d1o_1	13	d1p_1	20	d3pp_2	27	d3p_3
7	d2o_1	14	d2p_2	21	d1o_2		

**Table A4:** Significant altered metabolic pathways. Raw *p* is the original *p* value calculated based on enrichment analysis; FDR is the *p* value after correction by FDR; Impact is the pathway impact value calculated from pathway topology analysis [36].

Pathways	Raw *p*	FDR	Impact
Aminoacyl-tRNA biosynthesis	1.26 × 10^−8^	1.01 × 10^−6^	0.11
Nitrogen metabolism	6.24 × 10^−7^	2.50 × 10^−5^	0.00
Glycine, serine and threonine metabolism	3.64 × 10^−5^	9.72 × 10^−4^	0.32
Alanine, aspartate and glutamate metabolism	9.03 × 10^−5^	1.81 × 10^−3^	0.47
Arginine and proline metabolism	1.19 × 10^−4^	1.90 × 10^−3^	0.36
Phenylalanine metabolism	1.88 × 10^−3^	2.16 × 10^−2^	0.17
Pantothenate and CoA biosynthesis	1.89 × 10^−3^	2.16 × 10^−2^	0.18
Pentose phosphate pathway	3.59 × 10^−3^	3.28 × 10^−2^	0.26
Cyanoamino acid metabolism	3.69 × 10^−3^	3.28 × 10^−2^	0.00
Cysteine and methionine metabolism	4.97 × 10^−3^	3.98 × 10^−2^	0.31
Glutathione metabolism	6.73 × 10^−3^	4.72 × 10^−2^	0.02
Citrate cycle (TCA cycle)	7.09 × 10^−3^	4.72 × 10^−2^	0.09
